# Relevance of Non-Targeted Effects for Radiotherapy and Diagnostic Radiology; A Historical and Conceptual Analysis of Key Players

**DOI:** 10.3390/cancers11091236

**Published:** 2019-08-23

**Authors:** Carmel Mothersill, Andrej Rusin, Colin Seymour

**Affiliations:** Department of Biology, McMaster University, Hamilton, ON L8S 4L8, Canada

**Keywords:** bystander effect, genomic instability, lethal mutations, radiotherapy, diagnostic radiology

## Abstract

Non-targeted effects (NTE) such as bystander effects or genomic instability have been known for many years but their significance for radiotherapy or medical diagnostic radiology are far from clear. Central to the issue are reported differences in the response of normal and tumour tissues to signals from directly irradiated cells. This review will discuss possible mechanisms and implications of these different responses and will then discuss possible new therapeutic avenues suggested by the analysis. Finally, the importance of NTE for diagnostic radiology and nuclear medicine which stems from the dominance of NTE in the low-dose region of the dose–response curve will be presented. Areas such as second cancer induction and microenvironment plasticity will be discussed.

## 1. Background History and Pathways Involved in Radiation-Induced Bystander Effects

### 1.1. Historical Introduction

Marcus Tullius Cicero, a prolific writer, orator, and conservative Roman senator, wrote a rather flowery Latin phrase in “On the Contempt of Death” roughly translating to “I am not ashamed to confess that I am ignorant of what I do not know” (*Tusculanae Disputationes)* in the years that encapsulated the death throes of the Roman Republic. The idea of accepting ignorance of a specific topic, rather than embracing dubious assumptions, before using rigorous scientific reasoning and experimentation to come to rational conclusions about a particular effect, was and continues to be an essential virtue in the field of radiation biology. Before discussing the contemporary field of radiation biology in relation to the likely relevance for radiation oncology and diagnostic radiology, it is important to understand the historical context of radiobiological research with regard to the different kinds of indirect radiation effects, the DNA-centric paradigm, and specifically, radiation-induced bystander effects (RIBE). Insight into these subjects in a historical context can provide invaluable knowledge for the study of the interaction between radiation and living systems.

Radiation biology is defined as the study of how ionizing radiation interacts with living organisms. The disciplines of radiation biology and molecular biology have been inextricably linked for most of the later part of the twentieth century when biologists began to work alongside physicists in the field. Up until about forty years ago, it was widely assumed that radiation acts on cells primarily through interacting with DNA [1]. This was a convenient model for determining the effects of ionizing radiation on cells, with researchers usually following a model involving direct damage to chromosomes, usually at high doses [1,2]. This so-called “DNA-Centric Paradigm” ensured that, for a time, radiation biology primarily focused on the effect of ionizing radiation on DNA directly, chiefly in the literature dating from the 1930s to the 1980s; as stated in Mothersill et al. [3], “genes were perfect cellular targets [of radiation] and with the discovery of DNA structure, everything seemed to fit and the “inconvenient truths (of radiobiological effects) were ignored”. 

### 1.2. A Brief Review of Indirect Radiation Effects 

Indirect effects of radiation are defined as a family of different yet connected radiobiological phenomena that act on living organisms not directly exposed to radiation [4]. The first reference to these effects can be traced back to 1905 [3,5] and began appearing in the literature under different names shortly thereafter. There are four main types of indirect effects, with each effect not being separate or entirely distinguishable from the others. The term “Abscopal Effect” meaning “out of field” refers to effects that appear to manifest in distant tissues from the site of exposure (see discussion in [3,6]). Abscopal effects are not always categorized as completely separate from RIBE [7]. Among all of the other effects, abscopal effects were the most studied in the early historical literature, partly because of their role in cancerous tumor radiotherapy [8,9]. The more historically ambiguous term “Indirect Effect” overlapped with abscopal effects in the past, but was mainly used to refer to more general indirect effects. The historical use and mere existence of this term challenged the commonly held notion that indirect radiation effects were exclusively the result of DNA alterations. One of the first references to this effect can be found in Jolles [10], which simply referenced a possible “diffusible substance” that might cause reactions in surrounding tissue following X-irradiation on a patch of skin. Around the time that the DNA-centric paradigm was most pervasive, the term “Clastogenic Factors” emerged; in hindsight, clastogenic factors are not completely distinguishable from contemporary RIBE either. Projects researching this subfamily mainly focused on chromosomal damage and potential generational effects (see discussion in [3]). “Radiation-Induced Bystander Effects” (RIBE) are generally defined as indirect effects of radiation that are consequent to the production of signals from irradiated cells that induce responses in unirradiated cells (see discussion in [5]). Probably the most important modern discovery among these different families of effects, RIBE research exhaustively perturbed radiobiologists’ preconceptions about the way that radiation interacts with cells, tissues, and living organisms, particularly in the 1980s when the idea was most controversial [1,2,3,11]. The discovery of RIBE was instrumental in the paradigm shift away from DNA in the 1990s [2,3,12,13,14].

Paradigm shifts in biology are often inescapable for researchers. The implications of a huge shift in understanding in any field is far-reaching, affecting researchers and oftentimes, the general public. The history of radiation biology and RIBE is made even more complicated with trenchant, ongoing discourse on models assessing risk for radiation exposure, such as the controversial Linear Non-Threshold Model (LNT), whose proponents cite extensive review, practicality, and ease of application [15,16,17], and whose critics refer to the need for a system-level approach incorporating non-human species, inherent uncertainty in low doses of radiation and indirect effects, misleading data that can lead to public hysteria, and extensive conflicting data [2,3,18,19,20]. This prolonged debate between radiobiologists and its effect on radiation protection policy revision necessitates thorough understanding of the equally important topics of direct and indirect radiation effects. Much of the history predating and following the 1990s paradigm shift is covered in Mothersill and Seymour [1,12], and most recently in Mothersill et al. [3]. A brief summary follows. 

### 1.3. Introduction to Modern Studies of RIBE

A series of early experiments by Jolles [10,21] determined that a bystander-like effect can be observed by examining surrounding tissue of an area exposed to X-rays. A paper by Parsons et al. [22] marked a watershed in the history of research into RIBE, although it was ignored until the start of this century [1]. Parsons et al. showed a reduction of cells in the sternal bone marrow of patients with chronic granulocytic leukemia who had been exposed to X-rays distant from the site of exposure. In the 1980s, a group of researchers began to challenge commonly accepted models of the action of ionizing radiation on tissues. Seymour et al. described so-called “lethal mutations” in 1986, Pampfer and Streffer described an in vivo type of genomic instability in 1989 and Kadhim et al. showed the appearance of delayed non-clonal chromosomal instability in 1992 [11,23,24]. The first modern report of bystander effects in non-irradiated cells was published in 1992 by Nagasawa and Little [25]. They examined the induction of sister chromatid exchanges in Chinese hamster ovary cells after exposure to very low fluences of alpha-particles; they noted that while less than 1% of nuclei were directly exposed, 30% of cells showed an increased frequency of sister chromatid exchanges. This research emerged during a time when many labs were beginning to question similar, DNA-centric assumptions that were present in radiation research previously (discussed in Mothersill and Seymour [1]).

Communication of the bystander signal has been studied extensively in the literature. Primarily, two forms of propagation emerged: soluble factors and gap-junction intercellular communication (GJIC). The former was studied in the literature through culture medium transfer experiments, the results of which imply that the effect can be propagated by soluble factors [26]. The latter form of communication is similar to the first; however, communication occurs between adjacent cells connected by gap junctions [27]. Recently, it was found that physical signals including UV biophotons can be released from irradiated cells that also induce bystander effects in surrounding cells [28,29,30,31]. Research into the communication of these signals is still ongoing in laboratories around the world. New research techniques point to a potential role for exosome communication and signaling mRNAs [29,32,33]. In addition to determining the mechanics of bystander signaling, of particular interest to researchers in the coming years may be the implications that bystander effects have in human disease, expanding on previous research in the field [34].

### 1.4. Biomolecular Pathways and Proteins of Interest

RIBE have been described in the literature as incredibly complex with respect to the sheer number of proteins, inorganic molecules, and cofactors believed to be involved. The molecules in question range from members of biologically ubiquitous kinase families to simple free radical species. In order to appreciate the interconnectedness of these molecules in our modern knowledge of RIBE, an elementary understanding of their involvement is required.

Nitric oxide and Reactive Oxygen Species (ROS) have been implicated as the source of the signal and in the propagation of RIBE for quite some time. Much of the suspicion surrounding radical chemical species stems from a number of studies linking them to DNA damage and the resulting indirect interaction with signaling proteins [35]. A study by Little et al. [36] using human fibroblasts and epithelial cells found that the suppression of NADPH oxidase and incubation with superoxide dismutase (SOD), an enzyme complex involved in free-radical production and a free-radical scavenger, respectively, led to substantial suppression of the bystander signal. Other researchers have corroborated these results with respect to SOD and NADPH [37,38,39]. Many groups focused on the production of intracellular ROS and their extracellular involvement as suspect in the radiation-induced bystander signaling cascade, noting the induction of micronucleus formation in bystander cells, regulation of the p53 pathway, and the effect on calcium channel fluxes [27,37,38,40,41,42,43]. Specifically, calcium influxes from the extracellular environment were assayed, which were linked to regulation of the bystander response in subsequent studies [43,44,45,46,47]. Other groups of researchers found that p53 wild-type cells exhibited radioresistance when growth medium was supplemented with nitric oxide [48], and that inhibiting nitric oxide in growth medium led to the abolishment of the bystander signal [49,50]. Research into the nitric oxide effect was expanded by later publications that discovered its positive effect in the propagation of the bystander signal [51,52]. 

Many proteins have been implicated in the bystander effect which can behave independently of ROS. Cyclogenase-2 (COX2) [53] and Serotonin [47,54,55] were also found to play a role in the bystander effect. NF-κB activity and its associated proteins, including COX2, were also proven to be involved in the bystander effect. Suppression of COX2 signaling activity was found to significantly reduce the bystander effect [53]. A later study by Zhou et al. [56] found that inhibition of NF-κB activation and scavenging of NO significantly decreased mutation frequency in bystander cells with both functional and nonfunctional mitochondria. They also found that the activity of NF-κB and associated proteins, specifically COX2 and inducible nitric oxide synthase (iNOS), were significantly lower in mitochondrial deficient cells. Some studies have also been published on the involvement of various cytokines, such as tumor necrosis factor alpha (TNFα) and interleukin 8 (IL8), which may act upstream of NF-κB and COX2 activation [57,58,59,60,61]. However, it is important to note some of the nuances in the former gene expression assay; common p53-regulated radiation exposure genes were expressed in elevated levels only in the directly exposed cultures, genes regulated by NF-κB, including the IL8 and COX2-coding genes *CXCL8* and *PTGS2*, showed no differential expression between directly irradiated cells and bystander cells immediately after irradiation, and in time-course analyses, genes controlled by NF-κB responded with waves of expression over the course of twenty-four hours. Another important cytokine that was thought to be the source of the intercellular propagation of the bystander signal is transforming growth factor beta (TGFβ). A study found that increased intracellular TGFβ corresponded with an “increased ROS bystander response” [62]. Previous studies indicated that increased TGFβ as a result of bystander signal exposure in human fibroblasts also resulted in increased intracellular and extracellular ROS, mainly in the form of hydrogen peroxide [41,63,64,65,66].

Broader pathways have also been investigated in RIBE research. Two of the most researched are the classical MAP kinase signaling pathway that involves MEK1 and MEK2/ERK [36,45,67,68], and the JNK/p38 kinase pathway [36,69]. Inhibition of ERK phosphorylation was found to repress the bystander response in the same study that found similar effects after COX2 suppression [53].

Mitochondria have been the subject of extensive study in RIBE research; consequently, the topic of mitochondrial dysfunction can causally be linked to proteins associated with mitochondria that are connected to RIBE [12,56]. One study that looked into the effects of mitochondria determined that mtDNA-depleted bystander cells that were also treated with respiratory-chain inhibitors had attenuated signs of DNA damage, which led researchers to believe that mitochondria and ROS play an important role in bystander effects [70]. Numerous other studies have reported the importance of mitochondria in the bystander effect [46,70,71,72,73,74,75], which can also be linked to the late p53 apoptosis pathway by extension.

**Figure 1:**
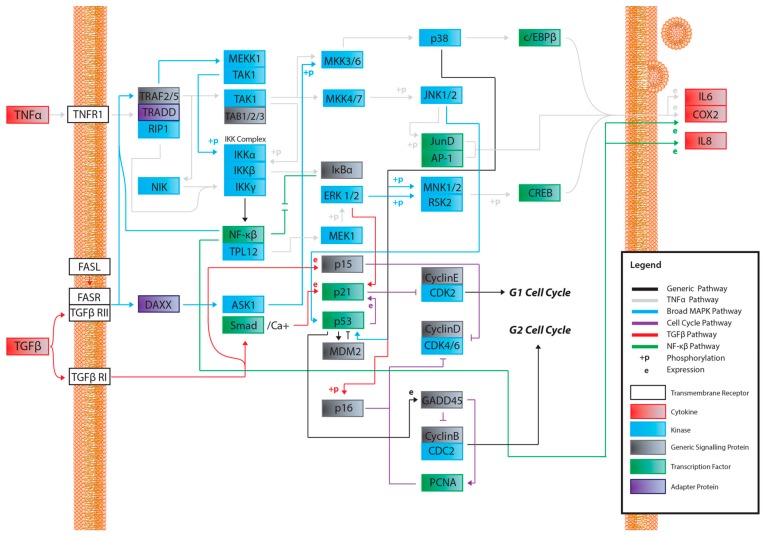
A map of potential pathways and their potential interactions activated by a soluble factor- radiation-induced bystander effect (RIBE) signal. While not entirely inclusive, this map was constructed to be displayed as a working reference point for current and future research. The pathways outlined in the legend have been identified as candidates for RIBE-intracellular and extracellular signalling. The following Figure 2, Figure 3, Figure 4 and Figure 5 are entirely derivative of this one and attempt to more closely showcase potential signalling within each pathway. Black lines or “generic pathways” represent interactions belonging to the other four pathways that could not be specified in this figure for clarity.

**Figure 2:**
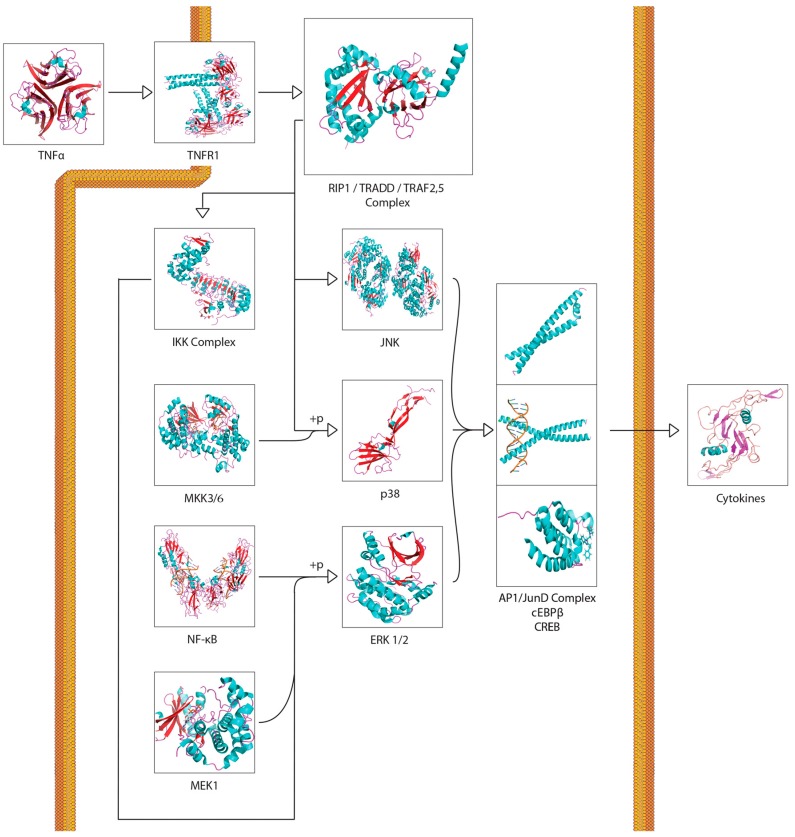
A simplified TNFα signalling transduction pathway. Activation of this pathway could lead to propagation of the RIBE signal through TNFα recognition and subsequent cytokine release.

**Figure 3:**
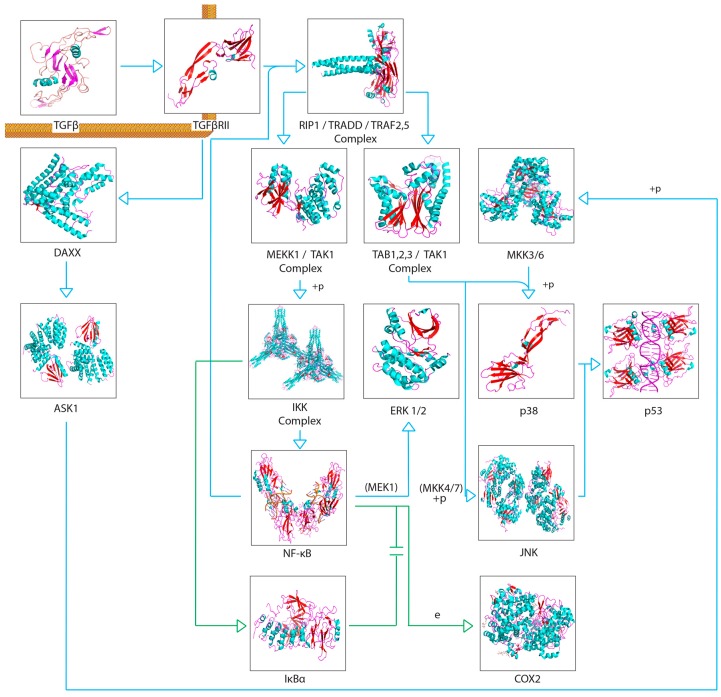
The classical MAPK pathway and a simplified NF-kB pathway. This pathway is of particular interest because of TGFβ’s ability to induce signalling through receptor recognition and signal amplification. The pathway is also of interest because of its endpoints, namely activation of p53 and various cytokines.

**Figure 4:**
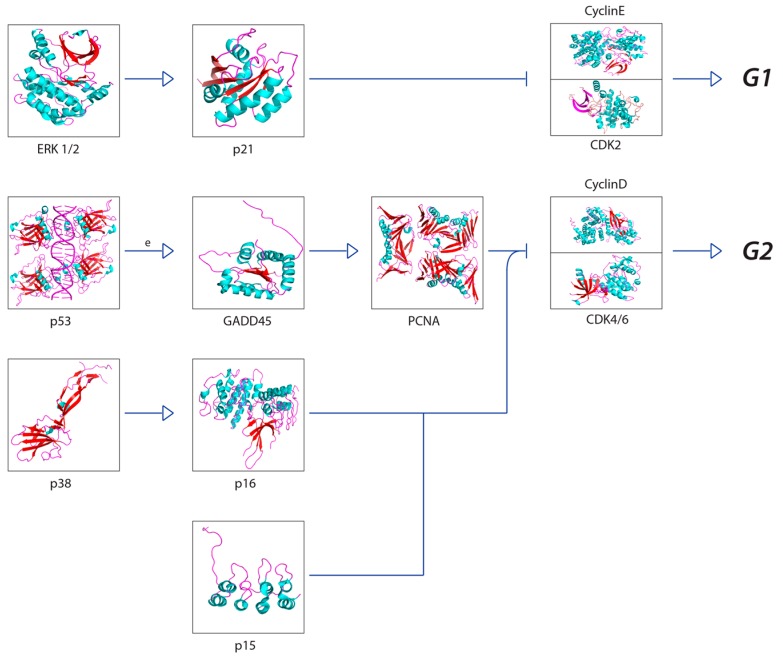
The simplified “cell cycle arrest pathway” downstream of p53 activation. This pathway ties in with the classic MAPK pathway and, therefore, TGFβ. This pathway also shows how p53 activation can lead to the expression of p21 and cell cycle arrest. It is important to note that p53 has many functions, and expression can also lead to apoptosis in other circumstances. The protein p21 is also involved in inhibition of other cyclin kinases and subsequent M-phase arrest (not shown) [59,60,61].

**Figure 5:**
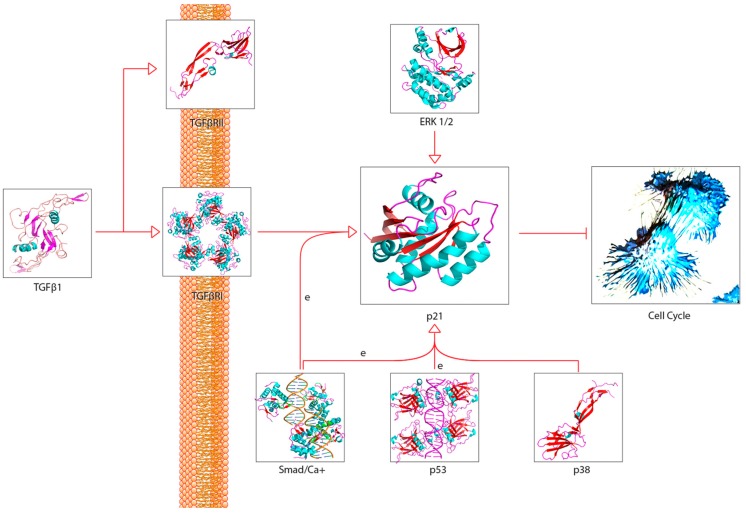
A simplified TGFβ pathway leading to p21 expression. While p21 can be expressed through different pathways such as these, it is important to quantify which pathways affect p21 expression and activation the most in the RIBE response. This pathway is intrinsically connected to the TNFα, MAPK, and cell cycle pathways as an endpoint for intracellular signalling.

A recurring theme in RIBE research is that the protein pathways and their intracellular and extracellular effects, such as ROS production, seem to overlap in one way or another. This is especially apparent when scouring for references to these proteins outside of the field of radiation biology. For example, NF-κB is thought to regulate expression of COX-2 [57], but NF-κB also acts downstream of a TNFα-activated cascade, leading to either proliferative, inflammatory, or anti-apoptotic events in mammalian cells [59,60,61,76,77]. In the broad MAPK/p38 pathway, a TNFα signaling cascade can lead to the phosphorylation of p38 (MAPK14) by MKK3, while upstream from this phosphorylation, TRAF2 can act on MEKK1, leading to the activation of NF-κB [59,60,61,78,79,80,81,82]. In the same general MAPK pathway, TGFβ binding to TGFβR—its associated cell membrane receptor—can lead to the downstream indirect phosphorylation of p38 (MAPK14) further down the cascade [59,60,61,83,84], which can lead to p53 activation [59,60,61]; activation of TRAF2, which is involved in the TNFα pathway, can lead to the same effect [59,60,61,85]. Another example, this one within the field of radiation research from the same group first reporting on GJIC, found that NADPH oxidase increases the accumulation of p53 and p21 and promotes micronucleus formation [38]. DNA damage was noted in directly irradiated fibroblast cultures, as well as Ser15-phosphorylated p53 accumulation. The group also found that SOD attenuated induction of p21 in bystander cells, showing that the downstream p21 is a stress response effector and that this was most likely due to the presence of ROS. No increase was detected in directly irradiated cells incubated with SOD; however, SOD introduction resulted in inhibited NF-kB. All these pathways are available in the Supplementary Materials provided and summaries of salient cascades are available as figures (Figure 1, Figure 2, Figure 3, Figure 4 and Figure 5). While this overview is likely not representative of all the potential interactions involved in RIBE nor their sequence, the importance of uncovering the inner workings of one particular pathway involved in RIBE while insisting on adhering the proverbial “bigger picture” is another virtue that appears to be indispensable in radiation biology. 

### 1.5. The Importance of the p53 Pathway

P53 is a protein known colloquially among molecular biologists as “the master switch” in controlling cell fate. In radiation biology and elsewhere, it is also known as “the guardian of the genome” which directs cells into apoptosis or stalls the cell cycle in G1 to allow for DNA repair. It is also known to be mutated in over 50% of human tumors [86]. It can be linked downstream or upstream of virtually all of the aforementioned proteins and inorganic molecules, including NF-κB, various proteins in the MAPK pathway, TGFβ, TNFα, COX2, NADPH oxidase, NO, ROS, IL6, IL8, and p21; such research has been conducted by various groups [30,87,88,89,90,91,92,93,94,95,96,97,98,99,100]. The omnipresence of p53 pathway research can be attributed to p53’s position in potentially innumerable signaling cascades that determine the fate of living cells, controlling such processes as cell cycle arrest, senescence, DNA repair and damage prevention, and apoptosis [101].

Studies have shown that the status of p53, both at the corresponding gene and post-translational level, greatly affects how cells respond to bystander signals. MDM2 is a p53-specific ubiquitin ligase that directly antagonizes p53, limiting its growth-suppressive function in unstressed cells [102]. P53, p21, and MDM2 were significantly modulated in bystander human fibroblast and epithelial cells, with the signals leading to this effect transmitted through GJIC [36]. As stated earlier, the bystander response was suppressed with incubation with SOD as well as an NADPH oxidase inhibitor, suggesting that RIBE can be transmitted by ROS. A 2–4-fold increase in the phosphorylation levels of JNK, ERK1/2, and a few other proteins was also observed [36]. At the time, it was hypothesized that this effect occurred as a direct result of DNA damage. Mothersill et al. [103] found that HCT116 cells with either null or wild-type p53 expression had different responses to the bystander signal. They showed that both null and wild-type cell lines could produce a signal, while only the wild-type cell lines could respond to the signal from either cell line. A study in 2015 expanded on this effect, finding that p53 wild-type HTC116 cells developed premature senescence in both directly exposed cells and in bystander cells [104]. The group also found that both directly exposed and bystander p53 null cells died primarily through apoptosis, that IL6 and IL8 expression were differently generated by both cell lines, that NF-κB was primarily activated in p53 wild-type cells that had been directly exposed, and that NF-κB was also mobilized significantly in null bystanders only.

P21 is a protein associated closely with p53 and has many roles in human cells. P53, p21, and their downstream proteins, among others, are also closely associated with the cell cycle. Interestingly, some studies showed that p53 and p21 were down-regulated along with concurrent upregulation of CDC2 and PCNA in bystander cells [105]. This is a point of interest because CDC2 inactivation is implicated in G2 arrest and PCNA is part of an extended signaling cascade that promotes S-phase proteins, with one study suggesting that p21 binding to PCNA can result in cell cycle arrest in p53-deficient cells [106,107,108,109]. This could indicate that exposure to the bystander signal can result in cell cycle arrest at various checkpoints.

HFL1 cells irradiated with X-rays show a senescence-like phenotype and phosphorylation of p53 at Ser15; this occurs concurrently with accumulation of p53 in intracellular space, followed by the induction of p21 and p16 [110]. Suzuki et al. [110] also determined that senescence-like growth arrest was dependent on p53 expression by using p53-null cells exposed to direct irradiation, presumably due to the effect on the p21 pathway and others. TGFβ is linked to cell cycle arrest by allowing the transcription of *CDKN2B,* the gene coding for p15, downstream of TGFβ’s expression [59,60,61]. Iyer and Lehnert [62] found that direct exposure of HFL1 cells to 1 cGy of alpha particles resulted in elevated levels of p53 and a decrease in CDC2. They also found that cells treated with medium from irradiated cultures on the first day of observation seemed to undergo a typical alpha-particle-induced cell cycle arrest; by the third day of observation, however, cell counts exceeded those from control groups. Curiously, the addition of small doses of recombinant TGFβ decreased the p53/p21 response, while larger doses stimulated the p53/p21 response. Better plating efficiency was observed with smaller doses of TGFβ, while larger doses decreased plating efficiency and increased the p53/p21 response. They also stated that bystander cells left alone after a few days showed reduction in p53 expression and increased expression of CDC2, which, as stated previously, is involved in proliferation and cell cycle arrest downstream of p53 in the p53 pathway [59,60,61,111].

While reading through the literature, specifically the studies on the p53/p21/TGFβ pathway, a few questions began to emerge. All of the studies that were reviewed seemed to treat p21 as solely dependent on p53 expression (e.g., [62]), or as a reliable marker for the expression of a functional p53 protein (e.g., [30]). This was interesting because some sources report that TGFβ could stimulate the expression of p21 on its own through a separate pathway involving control of p21 at the transcriptional level. An older study noted that the *CDKN1A* promoter region, the gene coding for p21, may require TGFβ and associated Smad proteins for its transcription [112]. In addition to this, the SP3 transcription factor that regulates induction of the promoter is calcium-dependent [112,113] which could provide a potential link to the observation that an influx of calcium into a cell is required for the bystander response [44,45].

Considering this information, a few interesting questions can be asked about the role of p53 and its associated pathways in the bystander signaling mechanism. Are genes like p21 activated in direct irradiations and in bystander neighbors in WT cells? Furthermore, are genes like p21 activated in direct irradiations and in bystander neighbors in p53 null cells? This is interesting because p21 inhibits cyclin-dependent kinases, ultimately leading to G1 cell cycle arrest [106,114,115]. It is also intrinsically linked to TGFβ through a Smad2,3 and Smad4 complex [116,117,118] and could, therefore, be linked to the bystander response. If p21 expression is compromised in p53 null cells and knowing that TGFβ is required for the transcription of *CDKN1A*, could one potentially use TGFβ protein-supplemented medium to restore expression of p21 in p53 null bystander cells? Would this restoration of p21 rescue the ability of p53 null cells to respond to the bystander signal, primarily by entering cell cycle arrest? These experiments would be particularly insightful because we could discern if the bystander effect acts on the p21/p53/TGFβ pathway differently in p53 WT compared to nulls, and, in addition, determine whether this effect is different or the same with direct irradiation and the related bystander signal.

Some similar research was conducted in the past. Recent studies in radiation biology have shown that the status of p53 affects the presence of p21 [30], but this paper in particular assayed UV biophoton ejection, which is out of the scope of this introductory review—although the finding that the UV biophoton bystander effect may follow a similar route of intracellular propagation is fascinating in its own right. In any case, quite a few studies have demonstrated that the downstream expression of p21 may be important to activity of p53 [119,120]. A crucially important study was conducted by Iyer and Lehnert [111] that showed that TGFβ was implicated in the p53/p21 bystander response and that TGFβ can decrease or increase concentrations of p53/p21 depending on the concentration of supplemented TGFβ. However, they did not investigate the potential for TGFβ to rescue the expression of p21 with an endogenous null expression of p53. If it were shown that it could, then TGFβ, and by extension, potentially all cytokines salient to the study of RIBE, can behave in multi-faceted, yet similar ways to achieve the same endpoint with respect to RIBE. It would also shed light on the issue of the p53 wild-type/null disparity observed in RIBE studies; either it is reliant on p53 expression completely, or cells can compensate for the lack of p53 expression via a different pathway to achieve a similar cell-cycle arrest phenotype. There is a lot of evidence in the literature to suggest that p21 is heavily involved in radiation-induced cell cycle arrest. One older study found that p21 deficiency abolishes direct-radiation-induced cell cycle arrest [106]. Subsequent studies have confirmed this and have linked the effect primarily to G1 arrest after DNA damage and a requirement for p53 activity [106,107,108,109].

As the term “RIBE” is commonly used to refer to a set of related effects in tissue culture consequent to radiation exposure, complications may arise in researching pathways related to p53 and other proteins. Specifically, the experimental comparison of independent endpoints of RIBE, such as apoptosis, sister chromatid exchanges (SCE), micronuclei formation, and clonogenic cell survival, may lead to seemingly contradictory results if other factors like the DNA repair capacity of bystander cells are not considered. It has been reported [121,122,123,124,125] that cell proliferation, transcriptional activity, replication stress and DNA damage repair processes modulate susceptibility of cells to RIBE. These findings demonstrate an added layer of complexity to RIBE model systems and should be taken into account by researchers aspiring to originate an exhaustive model system in future RIBE research. 

### 1.6. Is There A “Unified Theory” to Describe the RIBE Biochemical Cascade?

**Figure 6:**
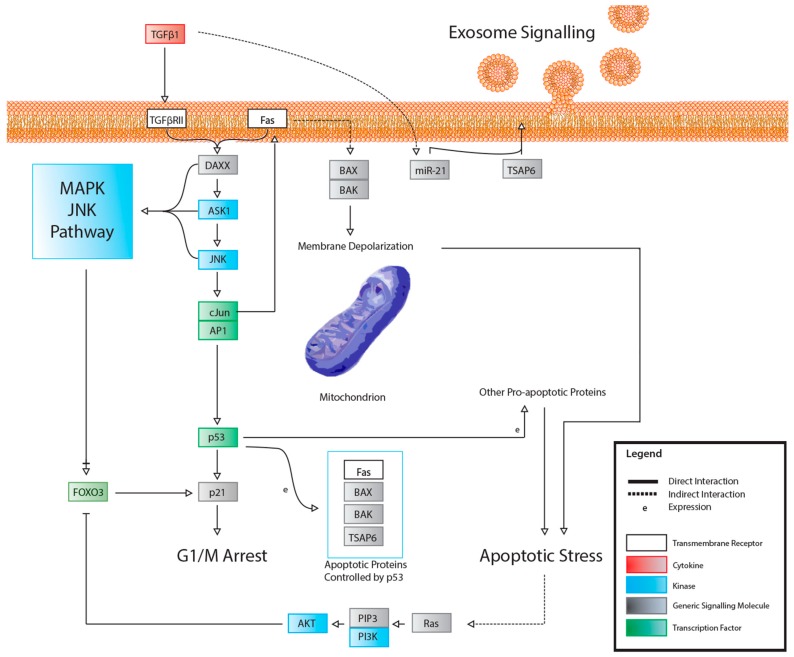
A hypothetical signalling pathway that illustrates how the various pathways in this review may be connected, which is partially informed by preliminary research.

Many of the studies reviewed look into the possibility of a unifying model for all bystander effects, but they generally separate different protein interactions from one another depending on the effect. Figure 1, Figure 2, Figure 3, Figure 4, Figure 5 and Figure 6 attempt to summarize and show the potential links between every protein and inorganic molecules mentioned in this introductory review. Although much of the content may be merely postulation with respect to RIBE, it is the hope of the authors that these visualizations of concepts covered in this brief introduction will help the reader further appreciate the interconnectedness of these pathways, rather than potentially persist in reductionism by examining a select few.

## 2. Non-Targeted Effects (NTE) in Tumours and Tumour Cell Lines Are Different to Those Seen Normal Tissues

### 2.1. Review of Evidence

Soon after the first publications in recent times relating to lethal mutations, genomic instability, delayed death or bystander effects, now known collectively as non-targeted effects or NTE [11,23,24,25,26,126], there was controversy in the literature and many reports of failure to find these unexpected consequences in non-irradiated cells or progeny of irradiated cells [127,128]. The initial confusion resolved somewhat when it was realised that all cell types did not show these effects [129,130] and that experimental conditions needed to be carefully controlled [131]. Later papers showed how complex the generation of NTE is with roles for p53 [132], serotonin [54,55,133,134,135], TGFβ [136,137,138], ROS [139,140,141], cell cycle phase [142,143] and many other factors (for reviews, see [3,144]). However, a broad division could be made depending on whether the cell line derived from a p53 mutant or null tumour cell like with low-dose radioresistance (a wide shoulder / high α/β ratio) where NTE, at least in the form of bystander effects (BE), were not seen and p53 wild-type, low-dose radiosensitive cell lines (small shoulder, low α/β ratio) where NTE were pronounced [145]. The particular situation of low-dose hypersensitivity/induced radioresistance (HRS/IRR) was particularly interesting since it seemed to be an anomaly until it was found that the BE were expressed in the HRS part of the dose–response curve but not after the dose response became IRR at higher doses [146,147]. These findings led to the generalisation that fast-growing cell lines which demonstrated radioresistance in the low-dose range, such as HT29 or PC3 [147], were less likely to show BE than slower growing radiosensitivive cells such as HaCaT or SW48 [147]. Later studies with tissues confirmed that tumour-derived explants and tumour-bearing animals or animals with tumour susceptibility had less pronounced BE than normal tissues or animals [148,149,150]. The situation with genomic instability (GI) was less clear, mainly because endpoints for GI included events resulting in increased death of progeny, such as lethal mutation or delayed death frequency [3,144] or gastrotrichus [151] but also included chromosomal instability endpoints where viable progeny were produced with increased potential for cancer development or transformation in vitro [152,153].

### 2.2. Possible Reasons/Mechanisms

**Figure 7:**
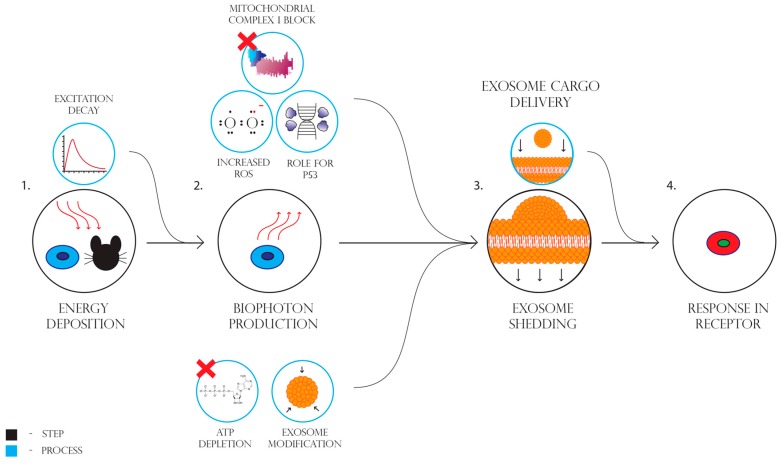
Steps and processes involved in the bystander effect as currently understood.

Once the phenomenology of NTE became more fully documented, attention turned to the mechanisms which could allow non-irradiated cells and distant progeny of recovered irradiated cells to display essentially the same endpoints (see Table 1). Initially, there was a focus on the use of separation techniques such as HPLC to try to determine the size and nature of the proposed molecule which caused NTE. These approaches proved unsuccessful. Attention turned to the response pathways which became the focus of mechanistic studies [13]. Cytokine activation was identified [154] and stress pathways downstream of ROS elevation were documented [45,155]. Apoptosis was found to be increased in the non-targeted cells [156,157], as were steps in the apoptotic pathway such as calcium flux, mitochondrial membrane depolarisation, caspase 3 release etc. [44,158]. DNA repair proficiency was also shown to be important with radiosensitive mutants (irrespective of the precise mutation), releasing stronger bystander signals than the wild-type parents [159,160,161,162,163]. This was later also shown in fish [164]. However, the nature of the signal from the irradiated cells remained a mystery. Breakthroughs came on two aspects of signal production in 2012 when it was shown that exosomes were released by irradiated cells into the culture medium [32,33]. Extracellular vesicles had been suggested early on as vehicles for bystander signals [165] but the molecular tools to analyse their contents were not widely available at the time. With the advent of ChIP technology and advanced proteomics techniques, screening for relevant proteins and miRNAs became possible [166]. The second breakthrough came when it was shown that irradiation of organic matter (shells, fruits or cells) led to biophoton emission in the UVA range and that there seemed to be a physical component to the initial bystander signal [31,167,168,169]. A series of papers [29,30] linked the photon emissions to the extent of the BE and implicated both p53 and exosomes in the mechanism (see Figure 7). The definitive experiments isolated exosomes and showed that exosomes from cells which received the UVA signal from irradiated cells without medium transfer, could by themselves produce a BE in never irradiated cells [29]. UVA alone was already known to produce BE from research in the 1990s [170,171]. Another piece of the puzzle fell into place with the linking of the UVA biophotons to a block of the activity of mitochondrial complex 1 [172]. This leads to depletion of cellular ATP levels and is the type of global issue that could explain many of the reported consequences of low-dose radiation exposure such as fatigue, reduced repair capacity and immune system compromise since these are all dependent of cellular energy availability [34,173]. Complex one block has been associated in the literature with UVA exposure and ROS elevation [174]. Currently, the race is on in many laboratories to profile exosomes in an attempt to further understand the mechanisms of transmission of bystander signals, although the evidence referenced above suggests that biophotons may be sufficient by themselves to induce both BE and GI. Regarding GI, the consensus is that this is driven at least in part by BE because GI can be triggered by bystander signals, as well as by direct irradiation, [175] and harvest of media from descendants of irradiated cells shows perpetuation of signal production in bystander cells [176].

### 2.3. Discussion of Relevance of Smoking and Other Lifestyle Factors

In addition to studying mechanisms associated with cellular genetic type, many studies have been done to look at the effects of environmental and lifestyle factors on the induction of NTE by radiation. These include the effects of heavy metals, organic pollutants, radium and tritium contamination, which confirm that both bystander signalling and GI can be modulated (usually increased) by concomitant exposure to a second stressor. In humans, the smoking history and in vitro treatment of human cells and explants with smoking specific nitrosamines prior to irradiation have been studied using a human explant model [177,178]. Radiation-induced BE was less toxic in terms of apoptosis induction in explanted tissues from smokers but that was associated with induction of anti-apoptotic proteins and suggests a pro-carcinogenesis rather than pro-apoptosis response to radiation in urothelium from smokers. Treatment of bladder urothelium from non-smokers with the specific nitrosamine 4-(methylnitrosamino)-1-(3-pyridyl)-1-butanol (NNAL), found in the urine of smokers, also induced this phenotype [179].

## 3. Relevance for Therapy—Possible Approaches to Enhance the Therapeutic Ratio

### 3.1. Inhibition of NTE Pathways in Normal Tissue

The paradox of NTE is that what may be good for an individual cell (e.g., not dying from the radiation dose) may be bad at the level of the population of cells or the organism, if living means carrying potentially carcinogenic damage. Also, given that NTE are more prevalent in normal cells than in tumour cells, the adverse effects of NTE in radiotherapy could be considerable. This area has been considered in detail by those studying “out of field” effects [180,181]. Reducing NTE in normal tissue is considered to be a potential novel target for improving radiotherapy outcomes. Those working on the complex one block in mitochondria which is implicated in many non-radiation-associated conditions such as chronic fatigue syndrome (CFS) have identified a number of places in the complex where specific activators or inhibitors of complex one could be targeted [182]. However, the situation may not be as simple as that because there are many reports of NTE-induced adaptive and protective responses acting at the population level [183,184] and NTE are seen by some as a mechanism for coordinating normal tissue level responses to harmful stimuli such as ionising radiation [87,88,89]. Clearly, it would not be wise to block such a mechanism. 

### 3.2. Stimulation of NTE Pathways in Tumour Tissues

The corollary of blocking NTE in normal tissues would be to try to stimulate NTE in tumour cell populations. To our knowledge, this approach has not been tried but could perhaps involve UVA/biophoton exposure concomitant with radiotherapy, or exposure to antioxidants during therapy to stimulate mitochondrial function. Possibly, if exosomes and their specific cargos could be harnessed, they could activate NTE in tumours as well.

## 4. Relevance for Diagnostic Radiology

### 4.1. Relevance of Low-Dose Dominance of NTE

There are two obvious areas of interest here given the enormous increase in the use of radiation-associated techniques in diagnosis of disease [185]. There is considerable controversy about whether any harm is being caused by such tests (for reviews, see [186,187]). The NTE-related concerns relate to the fact that NTE dominate the dose response at low doses and can be triggered by acute exposures as low as 2–3 mGy and increase until the NTE response saturates at about 0.5 Gy, at least in vitro [188,189,190]. There is no information about saturation or initiation doses in vivo or in humans but very early work by this group correlated low-dose radiosensitivity (then reported in terms of survival curve shoulder width or n value) with the burden of delayed lethal mutations [191,192]. This suggests that a retrospective analysis of human-derived cell lines, or samples where bystander signal information could be obtained, for example, the human skin series [193] or the RERF (Radiation Effects Research Foundation) blood samples from A-bomb survivors [194], might be interesting to correlate with the subsequent epidemiology. The two main NTE concerns are 2nd cancer induction, and microenvironmental plasticity due to genomic instability.

#### 4.1.1. 2nd Cancer Induction 

Cell transformation in vitro has been shown as an endpoint in non-targeted cells [194,195] and modelling of bystander effect impacts on the cancer induction dose response using linear-non-threshold approaches [196] have suggested that NTE may increase the chances of second cancer induction. Further evidence for a potential role of NTE in 2nd cancer induction after low-dose exposures comes from early data in the literature showing persistent expression of clastogenic factors, micronuclei or microsatellite instability in distant progeny of those exposed or in blood of those exposed several years earlier [197,198,199,200]. These data have been reviewed several times but not with respect to the possibility of NTE being involved in second cancer induction (for example, [3,201]). 

#### 4.1.2. Microenvironmental Plasticity

This refers to the ability of the microenvironment to change in response to changes in the system [202]. There is considerable interest currently in what is termed “cross-talk” between functional units in organs and support tissues such as endothelium, fibrous tissue and components of blood and endocrine systems [203]. Maintenance of a healthy microenvironment is critical to the control of function and to the abolition of pre-cancerous cells [204]. Induction of NTE signalling probably has multiple roles depending on other factors such as genetic or epigenetic makeup [205], environmental or lifestyle factors [206] or age [207], all of which can modulate the processes of NTE and the outcomes which may ultimately emerge. Key factors in defining the role of NTE in microenvironmental plasticity are the level at which the effects are of concern e.g., cell, organ, individual or population and the time over which adaptive or mal-adaptive instability has operated. The follow-up epidemiological studies on patients who have experienced low-dose medical diagnostic exposure should be an important source of information in this regard.

## 5. Conclusions

This review highlights areas in radiotherapy and diagnostic radiology where non-targeted effects of radiation may be important drivers of outcomes. Areas of most concern relate to the low-dose induction of genomic instability and to modulation of normal radiation response pathways by altered signalling due to bystander effects. The importance of context (environmental stressors, lifestyle, age and genetic background) are also discussed. Clearly, these processes could be involved in determining outcomes after radiation exposure (diagnostic or therapeutic) and should probably be further considered in radiation medicine.

## Figures and Tables

**Table 1:** Reported Effects.

Direct Irradiation Effects	Effects in Descendant Progeny and Neighbours *
Death	Death
Reproductive Failure	Reproductive Failure
Cellular Apoptosis	Cellular Apoptosis
Mitochondrial Defects	Mitochondrial Defects
Proteomic Changes	Proteomic Changes
Signalling Defects	Signalling Defects
Adaptive Responses	Adaptive Responses
Genetic Differences in Radiosensitivity	Genetic Differences in Radiosensitivity

* Persistent effects in descendant progeny that occur following no further irradiation and in cells neighbouring directly irradiated cells, however, the descendant progeny never directly exposed themselves.
